# Effect of hip dysplasia on the development of the femoral head growth plate

**DOI:** 10.3389/fped.2023.1247455

**Published:** 2023-10-16

**Authors:** Ningtao Ren, Zhendong Zhang, Yong Li, Ping Zheng, Hui Cheng, Dianzhong Luo, Jianli Zhang, Hong Zhang

**Affiliations:** ^1^ Department of Orthopedics, Fourth Medical Center of PLA General Hospital, Beijing, China; ^2^ Department of Orthopedics, Fuzhou No.2 General Hospital (Fuzhou No.2 Hospital), Fuzhou, China

**Keywords:** developmental dysplasia of the hip, femoral patient, skeletal immaturity, femoral head, growth plate

## Abstract

**Purpose:**

The purpose of this study was to observe whether developmental dysplasia of the hip (DDH) affects the development of the femoral head growth plate and to analyze the risk factors.

**Methods:**

We selected female patients aged between 11 and 20 years with unilateral DDH and unclosed femoral head growth plate (s). The selected patients underwent anteroposterior radiography of the hip joint to compare the degree of development of the femoral head growth plate on both sides and to identify risk factors that affect the development of the growth plate in the femoral head.

**Results:**

We included 48 female patients with unilateral DDH, with an average age of 14 years (range: 11.1–18.5 years) and an average BMI of 20.4 kg/m² (range: 15.5 kg/m²−27.9 kg/m²). Among them, 23 patients had earlier development of the femoral head growth plate on the affected side than on the healthy side, while the degree of development of the femoral head growth plate in 25 patients was the same as that on the contralateral side. When the Tönnis angle was greater than 29.5°C and/or the Reimers migration index was greater than 48.5%, there was a statistically significant difference in the acceleration of femoral head growth plate development.

**Conclusion:**

An abnormal relative position of the acetabulum–femoral head caused by DDH can accelerate closure of the femoral head growth plate in immature female patients. The risk factors are a Tönnis angle greater than 29.5°C and/or Reimers migration index greater than 48.5%.

## Introduction

The epiphyseal plate is a crucial structure during human growth and development, serving as the foundation of skeletal development (1). In long bones, it's a cartilaginous structure located between the epiphysis and diaphysis, regulating the growth of the bone shaft through endochondral ossification until the epiphysis and diaphysis are completely fused. Abnormal development or damage to the growth plate may lead to severe limb deformities and length discrepancies during bone growth. The growth of the epiphyseal plate primarily depends on the activity of cartilage cells (2). Genetics, race, nutritional status, hormone secretion, and external environmental factors are among the many factors that may affect the activity of epiphyseal plate cartilage cells. Mechanical loading is a significant external factor that can regulate the growth of epiphyseal plate cartilage. Usually, the growth plate can be affected by various complex forces, such as compression, tension, and shear forces. As stress increases, the shape of the epiphyseal plate changes as it adapts to the mechanical influences (3, 4).

The treatment of developmental dysplasia of the hip (DDH) is linked to the maturity of hip joint development (5–8). The degree of epiphyseal plate development is a marker of skeletal maturity. We can assess the maturity of the hip joint by the degree of development of the femoral head growth plate. The hip joint is a ball-and-socket joint composed of the femoral head and the acetabulum. The development of the femoral head growth plate is related to the normal head-acetabulum relationship. When the head-acetabulum relationship changes, the mechanical load on the femoral head will also change. Some studies have revealed that changes in hip joint mechanical loading patterns during growth can cause proximal femoral deformities (9, 10). This suggests that the development of the proximal femoral epiphysis is related to hip joint loading pattern. Hip joint loading pattern is related to hip joint structural development. DDH patients typically have an abnormal head-acetabulum relationship, resulting in a smaller contact area between the femoral head and the acetabulum, leading to abnormal stress on the femoral head growth plate. The mechanical regulation of endochondral ossification in the epiphyseal plate and the pathological mechanism of progressive skeletal deformities are closely related, especially when skeletal development is immature (11, 12).

Based on the above, we hypothesize that in DDH patients with immature skeletal development, the abnormal head-acetabulum relationship of the hip joint will cause changes in the development of the proximal femoral head growth plate.

## Patients and methods

### Patient selection

From January 2014 to December 2020, a total of 128 female DDH patients between the ages of 11 and 20 were selected from our center. The inclusion criteria were as follows: (1) unilateral hip dysplasia, the affected hip joints of the patients all met the criteria of Tönnis angle more than 10°, lateral center-edge angle less than 25°C, and incomplete dislocation; (2) all patients included in the study had standard pelvic radiographs, without obvious pelvic rotation; (3) the affected hip joints had either simple developmental dysplasia or subluxation, and at least one of the femoral head epiphyses had not yet closed.

The exclusion criteria were as follows: (1) patients with bilateral femoral head growth plate completely closed; (2) history of hip joint surgery; (3) other diseases affecting bone epiphyseal development, such as multiple epiphyseal dysplasia (MED) and Legg-Calvé-Perthes disease (LCPD); (4) endocrine disorders and neuromuscular dysplasia that affect bone epiphyseal development.

According to the development of the femoral head growth plate on the affected side, the patients were divided into the group with earlier development of the femoral head growth plate on the affected side (i.e., the femoral head growth plate on the affected side developed earlier than that on the contralateral side) and the group with unaffected development of the femoral head growth plate on the affected side (i.e., the femoral head growth plate on the affected side had no change compared to that on the contralateral side).

### Observation indicator

**Figure 1:**
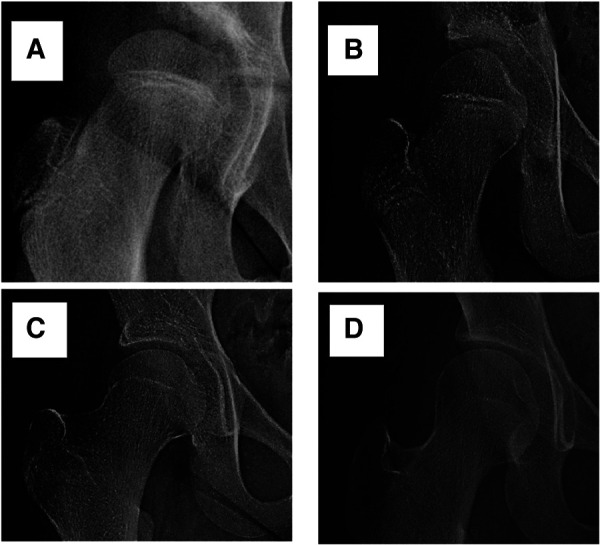
The developmental grades of the growth plate of the femoral head. Stage (**A**) (Grade 1): the beginning of the closure of the growth plate, with the chondrocyte gap narrowing and bone trabeculae visible. Stage (**B**) (Grade 2): partial closure of the growth plate (with less than 50% closure), usually starting from the middle and the chondrocyte gap between the trabeculae narrowing compared to Stage (**A**). Stage (**C**) (Grade 3): almost complete closure of the growth plate (with more than 50% closure), with some gaps still visible on one or both sides. Stage (**D**) (Grade 4): complete closure of the growth plate, with the residual or complete disappearance of the chondrocyte gap.

With reference to the female adolescents skeletal development indicators, based on the Tanner-Whitehouse 3 (TW3) method, the development of the femoral head growth plate was classified into four grades (see Figure 1) (13). Standard pelvic anterior-posterior radiographs were obtained using the Definium-6000 digital x-ray imaging system (GE-Healthcare) and standard x-ray imaging procedures. Two experienced pediatric orthopedic surgeons graded the degree of development of the femoral head growth plate on the PACS system, as well as measured the lateral CE angle, Tönnis angle, and Reimers migration index.

### Statistical methods

SPSS 26 statistical software (IBM, Armonk, NY, USA) was used for statistical analysis. Independent sample *t*-tests were performed on the age, BMI, lateral CE angle, Tönnis angle, and Reimers migration index of the early-developing group and the unaffected group. *P* < 0.05 indicates statistical significance. The risk threshold value was calculated through the ROC curve.

## Results

48 female patients with unilateral DDH were selected. Among them, 23 cases had earlier femoral head growth plate development on the affected side, while 25 patients had no affected femoral head growth plate development. The average age of the early femoral head growth plate development group was 13.9 years, while the average age of the unaffected femoral head growth plate group was 14.1 years (*P* = 0.783). The average BMI of the early femoral head growth plate development group was 20.5 kg/m², while that of the unaffected femoral head growth plate group was 20.3 kg/m² (*P* = 0.836).

**Figure 2:**
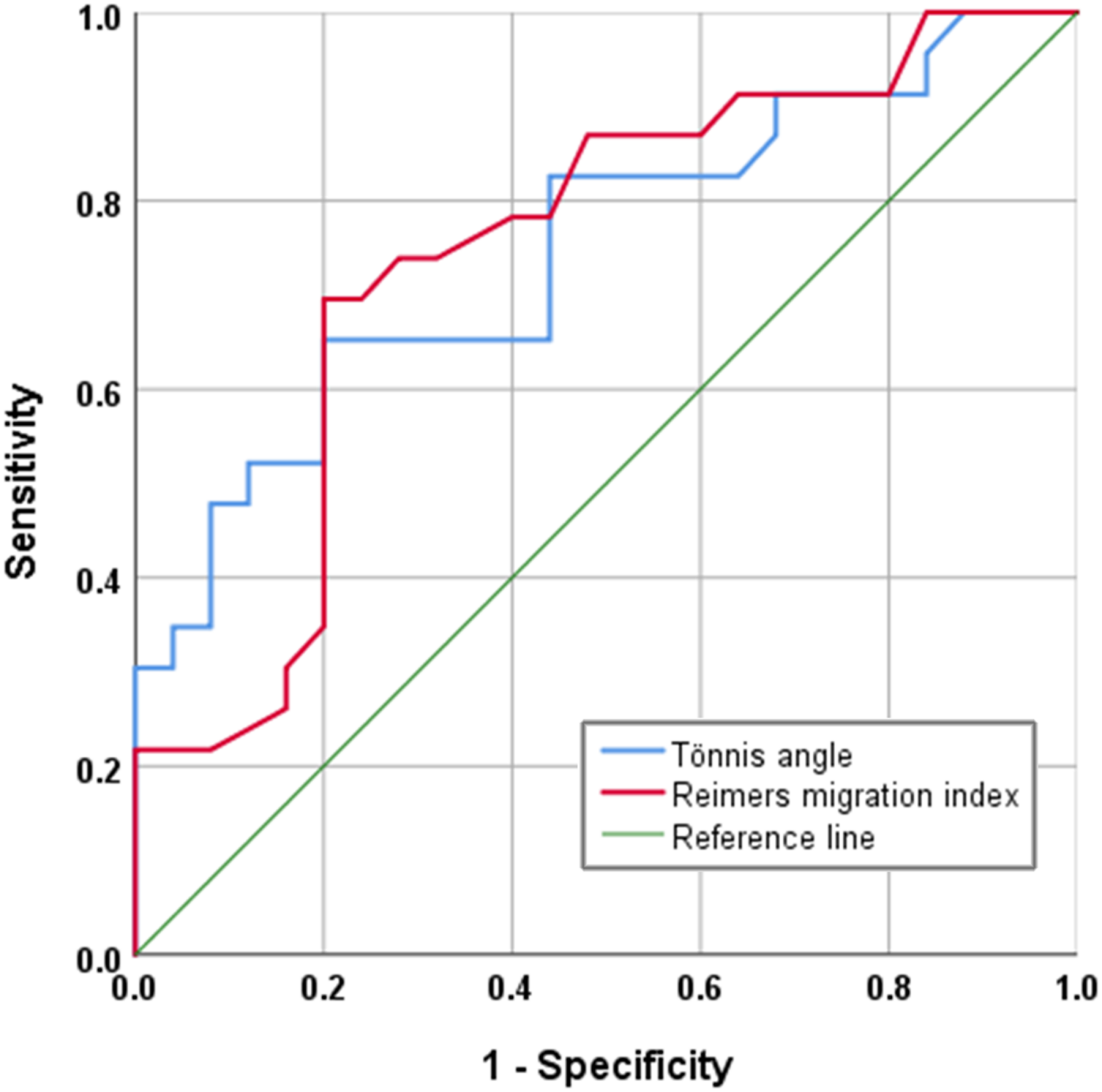
ROC curve. Tönnis angle AUC (Area Under Curve) is 0.748; Reimers migration index AUC (Area Under Curve) is 0.745.

**Figure 3:**
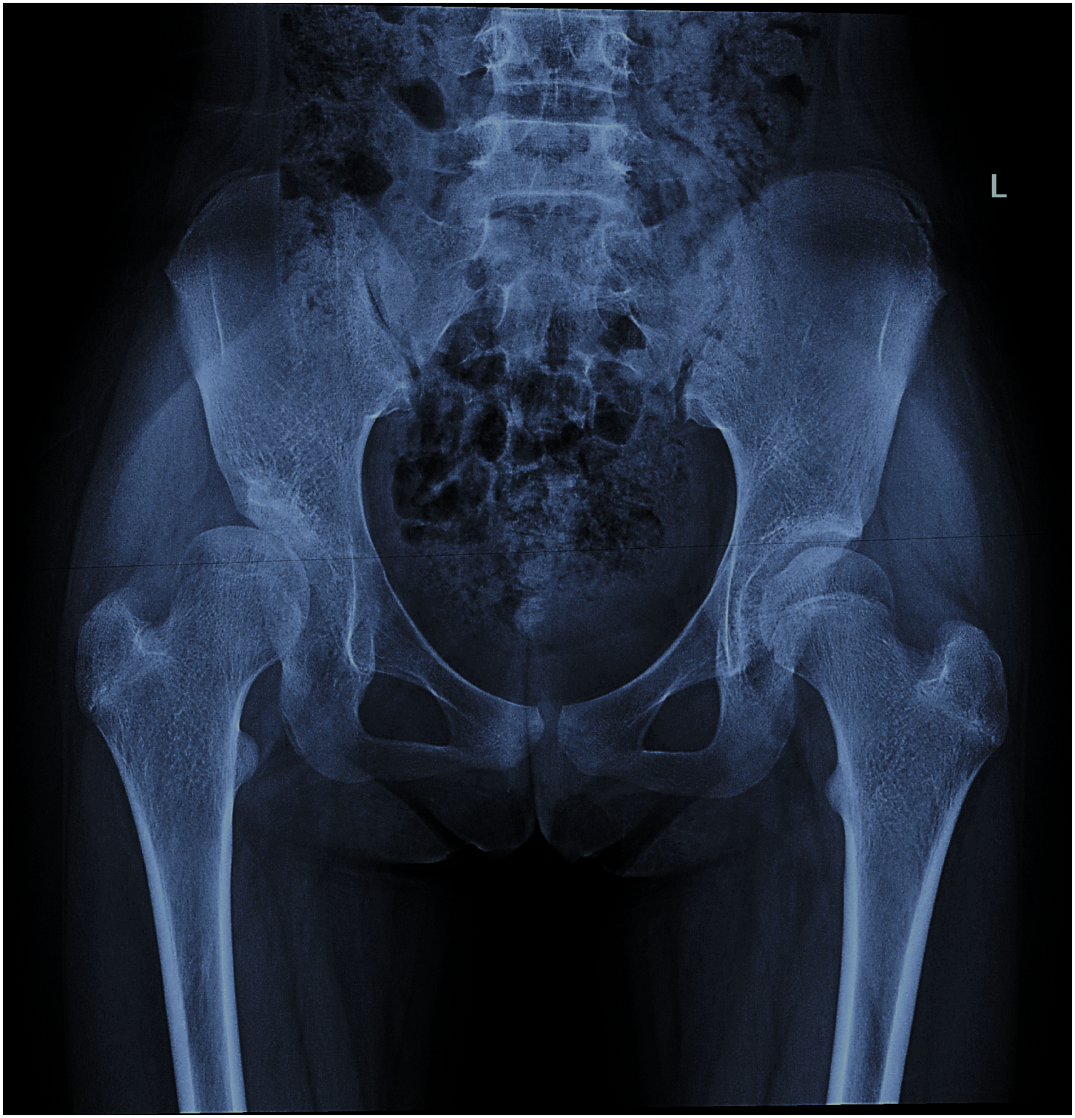
A 12-year-old girl with hip dysplasia on the right side, where the tönnis angle is 41.2°, and the Reimers migration index is 53%. The growth plate on the affected side closed earlier than that on the healthy side.

**Figure 4:**
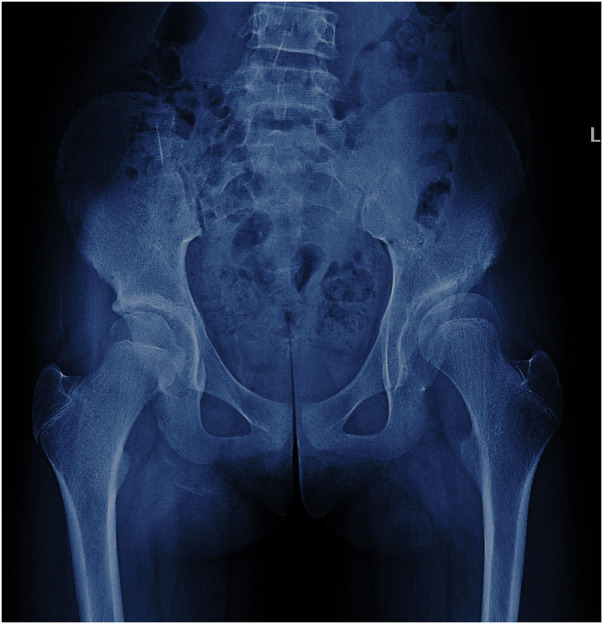
A 13-year-old girl with hip dysplasia on the left side, where the tönnis angle is 25.4°, and the Reimers migration index is 24%. The developmental status of the growth plate of the femoral head is consistent on both sides.

In radiographic measurement, the average lateral CE angle of the early femoral head growth plate development group was −6.1°C, while that of the unaffected femoral head growth plate group was −1.6°C (*P* = 0.099). The average Tönnis angle of the early femoral head growth plate development group was 33.8°C, while that of the unaffected femoral head growth plate group was 26.0°C (*P* = 0.002). The average Reimers migration index of the early femoral head growth plate development group was 50%, while that of the unaffected femoral head growth plate group was 41% (*P* = 0.003) (Table 1). By calculating the risk threshold value through ROC curve (Figure 2), when the Tönnis angle is greater than 29.5°C and/or Reimers migration index is greater than 48.5%, accelerated the development of femoral head growth plate (see Figures 3, 4).

**Table 1:** Compares the age, BMI, and imaging measurements of two groups.

Group	ED group	UD group	*P* value
Age (year)	13.91 ± 2.02	14.06 ± 1.85	*0* *.* *783*
BMI (kg/m^2^)	20.50 ± 3.72	20.31 ± 2.54	*0*.*836*
LCEA (°)	−6.05 ± 9.23	−1.64 ± 8.90	*0*.*099*
Tönnis angle (°)	33.81 ± 9.19	26.00 ± 6.81	***0***.***002***
RMI (%)	50.17 ± 10.03	40.88 ± 10.52	***0***.***003***

ED, earlier development of the femoral head growth plate; UD, unaffected development of the femoral head growth plate; LCEA, lateral CE angle; RMI, reimers migration index.

The bold values represent statistically significant differences between the groups.

## Discussion

The Hueter-Volkmann law (also known as the bone epiphysis pressure law) describes the relationship between bone epiphysis growth rate and the pressure to which they are subjected (14). Increased pressure on the epiphyses inhibits bone growth, while decreased pressure accelerates it. However, excessive pressure can impede normal epiphyseal growth. Mechanical stress can accelerate the formation of secondary ossification centers, regulate the entry of capillaries into cartilage ossification and growth plates, and affect the growth rate of growth plates at the cellular level. If the pressure on one side of the growth plate exceeds a certain level or with a high-pressure load, it can cause an increase in cell apoptosis. The abnormal mechanical environment affects the development of growth plates during immature skeletal development and is related to the pathological mechanism of progressive skeletal deformities (11, 12, 15–29).

Under normal circumstances, the hip joint only bears partial weight. However, when developmental dysplasia of the hip (DDH) occurs, the force surface can gradually shift to the edge of the acetabulum, and even show point-surface contact. This can significantly increase the pressure on the hip joint, up to 2–3 times that of normal. This change becomes more obvious as the Tönnis angle increases and the degree of lateral displacement of the femoral head increases. It can lead to occlusion of the lateral artery of the femoral head growth plate and affect the development of the growth plate. Clinical studies have also found that excessive stress can affect the growth and development of bone epiphyseal growth plates. Changes in hip joint load patterns during growth can cause proximal femoral deformities (30, 31). However, there were few studies on the development of growth plate of femoral head in DDH. Our research found that when the Tönnis angle was greater than 29.5°C and/or Reimers migration index was greater than 48.5%, the femoral head growth plate was in an unfavorable mechanical environment. This may cause accelerated closure of the femoral head growth plate in immature female DDH patients. This highlights the importance of early intervention.

The key to the treatment of children with DDH is to obtain concentric reduction (32). When the shape of the femoral head is not spherical, concentric reduction is impossible (33). The less circular the shape of the femoral head, the worse the treatment effect (34, 35). The shape of the femoral head is not only related to age but also to the growth environment around the femoral head growth plate. When the femoral head growth plate is in an unfavorable stimulation environment or there is a lesion, it can cause changes in the shape of the femoral head (35–38). This is also the principle of conservative treatment for DDH in infancy (39). In immature patients with skeletal development, when the mechanical environment of the femoral head growth plate improves, it can cause deformation of the femoral head shape (40–42). Therefore, early intervention is necessary when abnormal head-acetabulum relationships are occurred to prevent abnormal growth and development of the epiphysis and growth plates due to long-term abnormal mechanical stress, which can affect the postoperative effect. Early improvement of the mechanical environment of the femoral head growth plate can achieve good femoral head shape and good head-acetabulum matching.

The limitations of this study were that: (1) it was a preliminary study on the effect of abnormal head-acetabulum relationships on the development of the femoral head growth plate growth plate in female unilateral DDH; (2) the sample size was limited, and there was a lack of comparison between gender and normal adolescents. (3) Different rotational positions of the lower limbs maybe affect the the observation of growth plates. The subsequent studies were that: (1) determine whether a postoperative plasticity of the femoral head will occur when the Tönnis angle is greater than 29.5°C and/or the Reimers migration index is greater than 48.5%; (2) increase the sample size and comparisons between genders. (3) To compare the development of femoral head growth plate between DDH and normal adolescents.

In conclusion, our findings demonstrate that the development of femoral head growth plates in immature female DDH patients and found that DDH can cause adverse changes in the mechanical environment of the femoral head growth plate, which can lead to early closure and may have a negative impact on the later treatment. The risk factors are a Tönnis angle greater than 29.5°C and/or Reimers migration index greater than 48.5%.

## Data Availability

The original contributions presented in the study are included in the article/Supplementary Material, further inquiries can be directed to the corresponding author.
